# The potential of stable carbon and nitrogen isotope analysis of foxtail and broomcorn millets for investigating ancient farming systems

**DOI:** 10.3389/fpls.2022.1018312

**Published:** 2022-10-19

**Authors:** Yu Dong, Xiaoguang Bi, Rubi Wu, Eric J. Belfield, Nicholas P. Harberd, Bent T. Christensen, Mike Charles, Amy Bogaard

**Affiliations:** ^1^ Institute of Cultural Heritage, Shandong University, Qingdao, China; ^2^ School of Archaeology, University of Oxford, Oxford, United Kingdom; ^3^ Department of Plant Sciences, University of Oxford, Oxford, United Kingdom; ^4^ Department of Agroecology, Aarhus University, Tjele, Denmark

**Keywords:** foxtail millet, broomcorn millet, stable isotopes, carbon, nitrogen, charring, farming, archaeology

## Abstract

Foxtail and broomcorn millets are the most important crops in northern China since the early Neolithic. However, little evidence is available on how people managed these two crops in the past, especially in prehistory. Previous research on major C_3_ crops in western Eurasia demonstrated the potential of stable carbon and nitrogen isotope analysis of charred archaeobotanical remains to reveal the management of water and manure, respectively. Here, we evaluate the feasibility of a similar approach to C_4_ millets. Foxtail and broomcorn millet plants grown in pots in a greenhouse under different manuring and watering regimes were analysed to test the effects of management on stable carbon and nitrogen isotope values of grains. Stable nitrogen isotope values of both millets increased as manuring level increased, ranging from 1.7 ‰ to 5.8 ‰ in different conditions; hence, it appears a feasible tool to identify manuring practices, in agreement with results from recent field studies. However, the two millets exhibit opposing trends in stable carbon isotope values as watering level increased. The shift in stable carbon isotope values of millets is also smaller than that observed in wheat grown in the same experimental environment, making it difficult to identify millet water status archaeologically. In addition, we charred millet grains at different temperatures and for varying durations to replicate macro-botanical remains recovered archaeologically, and to evaluate the offsets in carbon and nitrogen isotope values induced by charring. We found that the stable nitrogen isotope values of foxtail millet and broomcorn millet can shift up to 1–2 ‰ when charred, while the stable carbon isotope values change less than 0.3 ‰. Overall, we demonstrate that stable nitrogen isotope values of charred foxtail and broomcorn millet seeds could provide insight into past field management practices, and both carbon and nitrogen isotope values can together inform palaeodietary reconstruction.

## 1 Introduction

Foxtail millet (*Setaria italica*) and broomcorn millet (*Panicum miliaceum*) are the most important crops in northern China since the early Neolithic (Liu et al., 2004; Zhao, 2004; Yang et al., 2012; Crawford, 2016; Zhao et al., 2020) and remain so today in some parts of China (e.g. northern Shanxi Province). They were not only staple foods for people, but also important feed for domestic pigs and dogs (Pechenkina et al., 2005; Barton et al., 2009; Chen et al., 2016). Broomcorn millet was also a widely used crop in late prehistoric Europe, introduced from East Asia in the 2^nd^ millennium BC (Motuzaite-Matuzeviciute et al., 2013; Filipović et al., 2020).

The way people manage their crops not only impacts crop yields and quality but also how people organize their labour and what they do in different seasons. However, little direct evidence exists on prehistoric management of millet crops. Isotopic analysis of charred archaeological remains can potentially provide insight on crop management strategies. Previous studies based on growing experiments, surveys in traditional farming communities, and analysis of archaeobotanical remains reveal that manuring of wheat and barley can lead to higher stable nitrogen isotope values, while water shortage can result in reduced carbon isotope discrimination (Araus and Buxo, 1993; Kanstrup et al., 2011; Bogaard et al., 2013; Wallace et al., 2013; Styring et al., 2016).

The existing literature largely focuses on crops in western Eurasia, while less is known regarding the effect of manuring and watering regimes on isotopic values of traditional crops of East Asia (An et al., 2015; Yang and Li, 2015; Lightfoot et al., 2016; Lightfoot et al., 2020). Previous studies focus on stable carbon isotope values of millets, while no research has investigated the interaction of manuring and watering on foxtail or broomcorn millet. Here we present results from a greenhouse pot experiment with foxtail and broomcorn millets grown at different manuring and watering levels, to assess the potential of stable isotope analysis of millet grains in reflecting these management parameters. When applying this approach to charred archaeobotanical remains, the premise is that charred grains retain their original isotopic signature or show a systematic offset in isotopic values. Some previous research demonstrates that charring effects on cereal isotope values depend on charring conditions (DeNiro and Hastorf, 1985; Yang et al., 2011; Nitsch et al., 2015). We examined changes in stable carbon and nitrogen isotope values of foxtail and broomcorn millets subjected to different well-defined charring temperatures and durations that replicate grains recovered archaeologically.

## 2 Scientific background

### 2.1 The variability of stable carbon isotope values in C_4_ plants

Depending on their respective photosynthetic pathways, plants can be classified as C_3_, C_4_, or CAM (Hatch and Slack, 1970). Major C_3_ crops include wheat, barley, rice, and legumes, while maize, sorghum, sugarcane, and millets constitute important C_4_ crops. The stable carbon isotope values of C_3_ plants can vary considerably depending on environmental and genetic factors. In general, the stable carbon isotope values of C_3_ plants increase with decreasing mean annual precipitation (Kohn, 2010). In contrast, the isotopic composition among C_4_ plants varies less than that among C_3_ plants because the potentially large effect of fractionation by RuBisCO is suppressed in the semi-closed bundle sheath (O’Leary, 1988; Bowman et al., 1989). In addition, the p_i_/p_a_ ratio (the ratio of intercellular to ambient partial pressure of CO_2_) in C_4_ plants can remain fairly stable under a wide range of light, water and nutrient conditions (Wong et al., 1985b; Wong et al., 1985a; Wong et al., 1985c). Farquhar (1983) proposed a simplified model for C_4_ plants:


(1) $$\Delta^{13}C=a+(b_{4}+\Phi b_{3}-a)\frac{p_{i}}{p_{a}}$$

where a is the diffusional fractionation factor (4.4 ‰), b_3_ is the discrimination factor against ^13^CO_2_ by RuBisCO (30 ‰), b_4_ is the fractionation due to PEPC (phosphoenolpyruvate carboxylase enzyme, –5.7 ‰) and Φ represents the fraction of CO_2_ that leaks from the bundle sheath into the mesophyll. The term in Eq. (1) that is multiplied by p_i_/p_a_ can be either positive or negative depending on the leakiness factor Φ. C_4_ plants often (but not always) display opposite trends in Δ^13^C in response to changes in stomatal conductance and water stress in comparison to C_3_ plants (Williams et al., 2001; Dercon et al., 2006; Ellsworth and Cousins, 2016).

Foxtail and broomcorn millets are both C_4_ plants, yet they belong to two different biochemical subtypes: foxtail millet is an NADP-ME subtype, and broomcorn millet is an NAD-ME subtype (Hattersley, 1982). Previous studies suggest that the *δ* ^13^C values of foxtail millet are positively correlated with water availability (An et al., 2015; Lightfoot et al., 2020) and negatively correlated with latitude or light (Yang and Li, 2015). In addition, there can be significant variations in *δ* ^13^C values among different accessions (ca. 1.5–2 ‰) (An et al., 2015; Lightfoot et al., 2016). Little research has addressed broomcorn millet. Yang and Li (2015) found a positive correlation between the *δ* ^13^C values of broomcorn millet and precipitation, i.e. its Δ^13^C values correlate negatively with precipitation.

In addition to water status, the level of nitrogen nutrition may also affect leaf photosynthetic capacity. Condon et al. (1992) found that low nitrogen nutrition can lead to lower *δ* ^13^C values in wheat grains. In contrast, several studies found that increased nitrogen supply correlated with reduction in the *δ* ^13^C values of maize (Dercon et al., 2006; Pansak et al., 2007; Lasa et al., 2011). Nevertheless, no clear nitrogen effect on the carbon isotopic discrimination in maize was found under an optimum water regime (Dercon et al., 2006).

### 2.2 The variability of stable nitrogen isotope values in C_4_ plants

Plant stable nitrogen isotope ratios reflect their nitrogen source (atmospheric nitrogen for N_2_-fixing plants or nitrogenous compounds such as ${\text{NH}}_{4}^{+}$ and NO_3_^-^ for non N_2_-fixing plants) and any fractionation during nitrogen uptake and assimilation (Evans, 2001). For non N_2_-fixing plants, the *δ* ^15^N values of plants largely depend on that of soils; plant nitrogen should have *δ* ^15^N values similar to, or lower than, that of the nitrogen in soil (Heaton, 1987). A wide variety of environmental factors can influence the *δ* ^15^N values of plants. Mean annual precipitation is found to be negatively related to foliar *δ* ^15^N values in several studies (Handley et al., 1999; Amundson et al., 2003). Investigations of traditionally farmed fields have also confirmed that barley grown at more arid sites has higher *δ* ^15^N values (Styring et al., 2016). Additionally, foliar *δ* ^15^N values are positively correlated with temperature, and warmer ecosystems are characterized by higher *δ* ^15^N values than colder ecosystems (Martinelli et al., 1999; Amundson et al., 2003). It is hypothesized that the higher soil *δ* ^15^N values of warmer areas is due to increased mineralization of soil organic nitrogen and gaseous losses of ^15^N-depleted nitrogenous compounds (Handley et al., 1999). Few studies have examined variability in stable nitrogen isotope values in C_4_ plants. Lightfoot et al. (2016) found significant variation (up to 6 ‰) in *δ* ^15^N values among different accessions of foxtail millet grown under the same conditions.

In addition to natural factors, agricultural practices can also have an impact on the *δ* ^15^N values of plants. Many studies have demonstrated that plants receiving animal manure have higher *δ* ^15^N values than unmanured plants (Szpak, 2014). Recent studies have shown that the *δ* ^15^N values of C_4_ plants (pearl millet, foxtail millet, and broomcorn millet) also increase with the application of manure (Fraser et al., 2013; Styring et al., 2019; Christensen et al., 2022). In addition, the type and amount of manure applied and the frequency of application all affect plant *δ* ^15^N values. Plants fertilized with seabird guano have the most elevated *δ* ^15^N values, followed by fertilization with pig manure, then cattle manure; plants fertilized with poultry manure have the least elevated *δ* ^15^N values (Choi et al., 2002; Bateman and Kelly, 2007; Szpak, 2014). However, the plant nutritive value of nitrogen in manure and the effect of manuring on plant *δ* ^15^N values depends not only on animal species but also on the composition of animal diets and the conditions for manure storage before application (Szpak, 2014).

Burning/shifting cultivation can also affect plant *δ* ^15^N values. For example, the *δ* ^15^N values usually increase in the first few years after burning (Szpak, 2014). In shifting cultivation, land parcels are usually left fallow for several years and then burned just prior to cultivation, hence high *δ* ^15^N values are also expected in shifting cultivation. Tillage is unlikely to change plants *δ* ^15^N values significantly (Selles et al., 1984).

Water availability can also affect the *δ* ^15^N values of plants. Lightfoot et al. (2020) found a positive correlation between *δ* ^15^N values of foxtail millet and the amount of water applied after excluding waterlogged plants. Reid et al. (2018) found similar patterns in pearl millet *Pennisetum glaucum*, in which *δ* ^15^N values increased as cumulative annual precipitation rose. However, Sanborn et al. (2021) suggested that water availability had little effect on the *δ* ^15^N values of *Pennisetum glaucum*. Similar observations were made by Swap et al. (2004) for C_4_ vegetation in southern Africa. On the other hand, Murphy and Bowman (2009) found a negative relationship between water availability and the *δ* ^15^N values of C_4_ plants. Similar information on broomcorn millet is currently lacking.

### 2.3 Charring effects

Charred seeds and wood are the most commonly preserved macroscopic plant remains in archaeological contexts. During charring, the starch and amino acids in grains react to form high-molecular weight melanoidins, which make grains resistant to microbial attack, enabling their preservation in the archaeological record (Braadbaart et al., 2004a; Braadbaart et al., 2004b; Braadbaart et al., 2004c; Styring et al., 2013). Under ideal conditions, charring of grains induces minimal morphological changes so they are still identifiable when excavated. However, charred grains can have slightly different isotopic compositions from fresh ones due to differential loss of compounds in the process of heating. For example, water and lipids that are volatile are lost at relative low temperatures (<150°C). Lipids usually have lower *δ* ^13^C values than the bulk, hence the residue ends with higher *δ* ^13^C values (Czimczik et al., 2002). At higher temperature, cellulose and other starches start to break down (>220°C) and volatilize (>300°C). Cellulose is isotopically heavier relative to the bulk, and so loss of cellulose and starches will lead to lower *δ* ^13^C values of the remnant (Czimczik et al., 2002). Depending on charring conditions and the initial composition of seeds, their carbonisotopic values may increase or decrease upon charring (Nitsch et al., 2015). Hence, experimental charring under well-defined conditions is needed to interpret any isotopic shifts in foxtail and broomcorn millets due to prehistoric charring. Previous studies have shown that well preserved grains with little distortion are produced at relatively low temperatures (220–240°C) (Charles et al., 2015). Simulating charring experiments of foxtail millet suggests that the offset of the *δ* ^13^C values of foxtail millet can range from 0.04 to –0.46 ‰ when charred at 50 to 300°C and the offset of the *δ* ^13^C values of broomcorn millet range from –0.02 to 0.49 ‰ (Yang et al., 2011). Previous experimental charring of wheat, barley, lentil and pea observed changes of *δ* ^13^C values of +0.11 ‰ (ranging from 0.003–0.22 ‰) (Nitsch et al., 2015). No information on the changes of stable nitrogen isotope values of foxtail and broomcorn millets is available. There is an 0.34 ‰ offset in the nitrogen isotope values between charred and uncharred pearl millet (Styring et al., 2019), comparable to the 0.31 ‰ average offset of charred wheat, barley, lentil, and pea (Nitsch et al., 2015).

## 3 Materials and methods

### 3.1 Growing conditions and sampling of greenhouse samples

Foxtail millet (*Setaria italica* L.), and broomcorn millet (*Panicum miliaceum* L. Kornberger) seeds were obtained from millets grown in the Askov long-term experiment (Christensen et al., 2022) and planted in pots (about 0.5 L) in late May and early June 2018 in the greenhouse at the Department of Plant Sciences, University of Oxford. Wheat (*Triticum aestivum*) seeds were planted together with millets served as a reference crop. Plant numbers were reduced in mid-June to leave only one plant per pot. The greenhouse setting was 16-h light/8-h dark photoperiod at 30°C (daytime)/28°C (nighttime) (irradiance 800 μmol of photons/m^2^ per second) with 80% relative humidity (RH). The plants grew under different combinations of manuring and watering levels, with 21 plants per species (**Table 1**). Different amounts of cattle manure that had been rotted down for 12 months were purchased from a local Oxfordshire farm and mixed with nitrogen-free clay granules (Profile Field & Fairway Soil Amendment, Rigby Taylor Ltd.) to achieve three manuring levels (1%, 5%, and 10% of organic matter in the mixed soil). Nitrogen-free clay granules, instead of regular pot compost mixtures, were chosen to eliminate the potential impact on nitrogen isotope values of mixed organic and mineral fertilisers. The percentage of organic matter was further confirmed by loss-on-ignition. The three manuring levels (1%, 5%, and 10%) represent low, high, and very high manuring practices respectively. Soils with 1% organic matter are commonly found in naturally occurring or low manuring level soils in northern China, soils with 4-5% organic matter are found in northeastern China; and soils with 10% organic matter are rare in the field (Cao et al., 2022; Zhang et al., 2022). Unfortunately, possibly due to insufficient mixing of growth medium and manure, many plants grown under 10% manuring failed. Hence, plants grown under this condition were excluded from further analysis. In addition, three watering levels were applied, i. e. 20% saturation, 60% saturation, and 100% saturation (correspond to 34 ml, 102 ml, and 170 ml of water supply per pot respectively). Water was added every 1–5 days when no water was observed in trays containing the pots. For each condition, three replicate pots were included, and their position randomized and rearranged daily to eliminate any impact of uneven growth conditions within the greenhouse. A nutrient solution (6 g CaHPO_4_ * 2H_2_O, 4.5 g K_2_SO_4_, 5 g MgSO_4_ * 7H_2_O, 1.5 g sequestered iron, and 0.5 g Hoagland’s no. 2 basal salt mixture, per 10 kg soil) was added to the pots every two weeks. The plants were harvested in mid-September when physiologically mature.

**Table 1:** Seven growing conditions of foxtail millet, broomcorn millet, and wheat at the greenhouse, Department of Plant Sciences, University of Oxford.

	1% manuring	5% manuring	10% manuring
**low watering level** **(20% saturation)**	1% manuring+low watering level	NA	NA
**medium watering level** **(60% saturation)**	1% manuring+medium watering level	5% manuring+medium watering level	10% manuring+medium watering level
**high watering level** **(100% saturation)**	1% manuring+high watering level	5% manuring+high watering level	1% manuring+high watering level

NA stands for not applicable.

A random sample of 20–30 grains was obtained from each plant for stable carbon and nitrogen isotope analysis; if a plant had multiple panicles, the whole set or half of the grains from each panicle (depending on the yield) were bulked before a sub-sample was taken for analyses. The palea and lemma were removed from the grains.

### 3.2 Millet charring experiments

For the charring experiments, dehusked foxtail and broomcorn millet grains were obtained from a local supermarket (grown in Weihai, Shandong, China). The study of Märkle and Rösch (2008) suggested that foxtail millet starts to become charred at 220°C under oxidizing conditions, while broomcorn millet needs slightly higher temperatures. Yang and colleagues (2011) found that foxtail and broomcorn millet start to become charred at 200°C, while heating at 250°C leads to swelling and some distortion, and destruction when heated at 300°C. The setup of the current study follows Nitsch et al. (2015), with charring temperatures at 215°C, 230°C, 245°C and 260°C, and heating durations of 4 hrs, 8 hrs, and 24 hrs. This provides 12 charring regimes altogether. Changes in seed morphology vary with charring conditions, with details discussed in Section 4.3. Three parallel samples were prepared under each condition. Three additional samples of both millets were dried at 50°C for 48 hrs to represent uncharred grains. Each sample consisted of 60 grains of foxtail or broomcorn millet. The charring experiment employed a muffle box furnace (SXL-1400C, Shanghai Jvjing) with seeds wrapped in aluminum foil and buried in sand, simulating conditions with restricted access of oxygen.

### 3.3 Isotopic analysis

Millets and wheat samples grown in the greenhouse were processed and analyzed at the School of Archaeology, University of Oxford, while charring effects on millets were analyzed at Shandong University. Specifically, grain samples from the growing experiment were homogenized using Spex 2760 Freezer/Mill before isotopic analysis. Milled samples were weighed into tin capsules and then run on a Sercon EA-GSL mass spectrometer at the Research Laboratory for Archaeology and the History of Art (RLAHA), School of Archaeology, University of Oxford. The *δ* ^13^C and *δ* ^15^N values were measured separately, using an internal alanine standard to obtain raw data and adjust for drift correction. In each run, additional calibration and check standards were run every 5–10 samples. An internal alanine standard and USGS 41 were used for the calibration of *δ* ^13^C values, and IAEA-CH6 was used as a check standard; the internal alanine and an internal seal standard were used for the calibration of *δ* ^15^N values and EMA B2159 sorghum was used as check standard.

Grain samples from the charring experiment were homogenized with an agate mortar and pestle after charring, and sub-samples of 1.5–3 mg were weighed into tin capsules. The samples were analyzed using a Thermo Scientific Delta V Advantage IRMS with a Flash 2000 HT Elemental Analyzer at the Joint International Research Laboratory of Environmental and Social Archaeology (JoInRLESA), Shandong University. The *δ* ^13^C and *δ* ^15^N values were measured together with dilution on carbon. An internal alanine standard was used to monitor drift correction. USGS 40, USGS 41a, and USGS 62 were used for calibrations and EMA B2159 sorghum was used as check standard.

The standard uncertainty of carbon isotope values is ±0.3 ‰ at both laboratories, while that of nitrogen isotope values is ±0.4 ‰ and ±0.2 ‰, respectively (Supplementary).

## 4 Results and discussion

### 4.1 Manuring and watering effects on the carbon isotope values of foxtail and broomcorn millets

**Figure 1:**
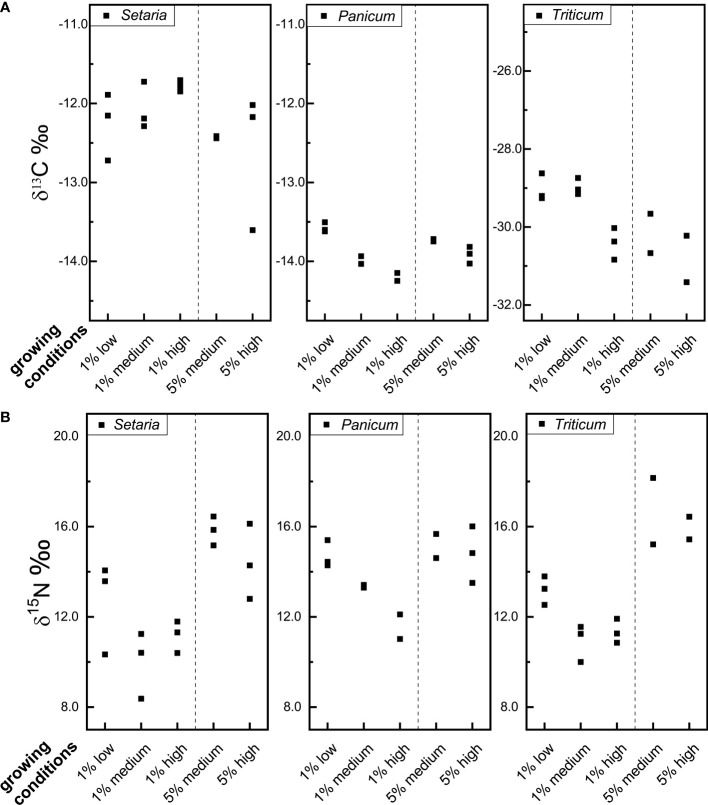
The carbon **(A)** and nitrogen isotope values **(B)** of foxtail millet (*Setaria italica*), broomcorn millet (*Panicum miliaceum*) and wheat (*Triticum aestivum*) grains grown under different manuring and watering conditions (for growing conditions, 1% and 5% are referring to the manuring levels, and low, medium, and high are referring to watering levels, for example, ‘1% low’ represents ‘1% manuring + low watering level’).

The stable carbon isotope values of *Setaria, Panicum*, and *Triticum* grains grown under different manuring and watering regimes are listed in **Table 2** and **Table S1**. The stable carbon isotope values of *Setaria* grains have an average of -12.3 ‰, -12.2 ‰, and -12.2 ‰ respectively for plants grown under low, medium, and high watering levels; the stable carbon isotope values of *Panicum* grains have an average of -13.6 ‰, -13.8 ‰, and -14.0 ‰ respectively; and the stable carbon isotope values of *Triticum* grains have an average of -29.0 ‰, -29.6 ‰, and -30.6 ‰ respectively (**Figure 1A**). The shift in carbon isotope values in response to different watering levels is smaller for *Setaria* and *Panicum* than for *Triticum*.

**Table 2:** The carbon and nitrogen isotope values of foxtail millet (*Setaria*), broomcorn millet (*Panicum*), and wheat (*Triticum*) grains grown under different manuring and watering levels.

		1% manure + L watering*, mean ± SD	1% manure + M watering*, mean ± SD	1% manure + H watering*, mean ± SD	5% manure + M watering*, mean ± SD	5% manure + H watering*, mean ± SD
*Setaria*	*δ* ^13^C	–12.3 ± 0.4 ‰ (n=3)	–12.1 ± 0.3 ‰ (n=3)	–11.8 ± 0.1 ‰ (n=3)	–12.4 ± 0.1 ‰ (n=3)	–12.6 ± 0.9 ‰ (n=3)
	*δ* ^15^N	12.7 ± 2.0 ‰ (n=3)	10.0 ± 1.5 ‰ (n=3)	11.2 ± 0.7 ‰ (n=3)	15.8 ± 0.6 ‰ (n=3)	14.4 ± 1.7 ‰ (n=3)
*Panicum*	*δ* ^13^C	–13.6 ± 0.1 ‰ (n=3)	–14.0 ± 0.1 ‰ (n=2)	–14.2 ± 0.1 ‰ (n=2)	–13.7 ± 0.1 ‰ (n=2)	–13.9 ± 0.1 ‰ (n=3)
	*δ* ^15^N	14.7 ± 0.6 ‰ (n=3)	13.4 ± 0.1 ‰ (n=2)	11.6 ± 0.8 ‰ (n=2)	15.1 ± 0.8 ‰ (n=2)	14.8 ± 1.3 ‰ (n=3)
*Triticum*	*δ* ^13^C	–29.0 ± 0.4 ‰ (n=3)	–29.0 ± 0.2 ‰ (n=3)	–30.4 ± 0.4 ‰ (n=3)	–30.2 ± 0.7 ‰ (n=2)	–30.8 ± 0.8 ‰ (n=2)
	*δ* ^15^N	13.2 ± 0.6 ‰ (n=3)	10.9 ± 0.8 ‰ (n=3)	11.3 ± 0.5 ‰ (n=3)	16.7 ± 2.1 ‰ (n=2)	15.9 ± 0.7 ‰ (n=2)

* L watering, M watering, and H watering corresponds to low, medium, and high watering levels respectively.

**Figure 1A** shows that the stable carbon isotope values of *Setaria* are positively correlated with watering level (though this is not statistically significant, Kruskal-Wallis test, **Table 3**), while the carbon isotope values of *Panicum* and *Triticum* are negatively correlated (not statistically significant, **Table 3**). The non-parametric Kruskal-Wallis test is chosen here because of the very small sample size (n=2 or 3). As discussed earlier (section 2.1), the carbon isotope values of C_3_ plants decrease with higher watering levels, while the carbon isotope values of C_4_ plants can increase or decrease in respond to elevated watering levels. The trend for *Setaria* in the current study is also found in previous studies on foxtail millet (An et al., 2015; Ellsworth and Cousins, 2016; Lightfoot et al., 2020) and pearl millet (Sanborn et al., 2021). The trend for *Triticum* confirms the expectations of a C_3_ plant (Wallace et al., 2013). The trend of *Panicum* observed in the current study is opposite to that found in the study by Yang and Li (2015). However, the study of Yang and Li (2015) relied on grains collected from widely different locations that varied not only in precipitation but also in latitude, altitude, and temperature. These parameters may all affect the carbon isotope values of *Panicum* grains. The plants grown in the current study vary in watering level only and are more likely to isolate the response of carbon isotope values to water availability in *Panicum* grains.

**Table 3:** Kruskal-Wallis test results of watering and manuring effects on the carbon and nitrogen isotope values of foxtail millet (*Setaria*), broomcorn millet (*Panicum*), and wheat (*Triticum*) grains.

	*Setaria*	*Panicum*	*Triticum*
**watering effects on carbon isotope values**			
1% manure, L watering *vs*. M watering *vs*. H watering	p=0.19	p=0.08	p=0.063
	(n=3,3,3)	(n=3,2,2)	(n=3,3,3)
5% manure, M watering *vs*. H watering	p=0.49	p=0.076	p=0.44
	(n=3,3)	(n=2,3)	(n=2,2)
**manuring effects on nitrogen isotope values**			
M watering, 1% manure *vs*. 5% manure	p=0.049	p=0.12	p=0.083
	(n=3,3)	(n=2,2)	(n=3,2)
H watering, 1% manure *vs*. 5% manure	p=0.049	p=0.083	p=0.083
	(n=3,3)	(n=2,3)	(n=3,2)
M and H watering levels combined, 1% manure *vs*. 5% manure	p=0.004	p=0.014	p=0.011
	(n=6,6)	(n=4,5)	(n=6,4)

L watering, M watering, and H watering corresponds to low, medium, and high watering levels respectively.

When comparing plants of the same watering level and different manuring levels, it is found that *Setaria* and *Triticum* grains grown with 5% manure have lower *δ* ^13^C values (0.3–1.2 ‰) than ones grown with 1% manure (**Figure 1A**, **Table 2**). This aligns with previous research on C_4_ plant maize where increasing nitrogen supply was found to reduce *δ* ^13^C values (Dercon et al., 2006; Pansak et al., 2007; Lasa et al., 2011). In contrast, *Panicum* grains grown with 5% manure have slightly higher *δ* ^13^C values (0.3 ‰) than plants grown with 1% manure (**Table 2**), which is opposite to the trend found in the Askov long term experiment field (Christensen et al., 2022). In general, however, the manuring effect on the *δ* ^13^C values of *Setaria* and *Panicum* is small and variable.

### 4.2 Manuring and watering effects on the nitrogen isotope values of foxtail and broomcorn millets

The stable nitrogen isotope values of *Setaria* grains range from 8.4 to 14.1 ‰ for plants grown with 1% manure, and from 12.8 to 16.5 ‰ for plants with 5% manure. Stable nitrogen isotope values of *Panicum* grains range from 11.0 to 15.4 ‰ when grown with 1% manure, and from 13.5 to 16.0 ‰ for plants with 5% manure. The values of *Triticum* grains range from 10.0 to 13.8 ‰ and from 15.2 to 18.2 ‰ for 1% and 5% manure, respectively (**Table 2**, **Table S1**, **Figure 1B**).

When comparing plants grown under the same watering level but different manuring levels (for example, 1% manure + medium watering *vs*. 5% manure + medium watering), it is clear that *Setaria*, *Panicum*, and *Triticum*, grown with 5% manure have much higher nitrogen isotope values than those grown with 1% manure (**Tables 2** and **3**). When medium and high watering levels are considered separately, statistical tests suggest the increase is not significant for *Panicum* and *Triticum*, but this is likely an artifact of the small sample size (only two plants, instead of three, survived in some conditions for *Panicum* and *Triticum*). However, when medium and high watering levels are combined, the increase is statistically significant for all three species (**Table 3**). In other words, all three crops have higher nitrogen isotope values as the manuring level increases. In addition, the magnitude of the shift in stable nitrogen isotope values caused by manuring is comparable for *Setaria* (shift of 5.8 ‰ and 3.2 ‰ with medium and high watering levels respectively), *Panicum* (shift of 1.7 ‰ and 3.2 ‰), and *Triticum* (shift of 5.8 ‰ and 4.6 ‰) (**Table 2**). This observation is also supported by complementary results from the Askov long term field experiment (Christensen et al., 2022).

When comparing plants with the same manuring level but different water levels, it is clear that *Setaria*, *Panicum*, and *Triticum* all have higher stable nitrogen isotope values as the watering level decreased (**Table 2**). The nitrogen isotope values increased 2.7 ‰, 3.1 ‰, and 2.3 ‰ for *Setaria*, *Panicum*, and *Triticum* respectively as watering level drops from high to medium or low in 1% manuring conditions, and increased 1.4 ‰, 0.3 ‰, and 1.8 ‰ respectively as watering level drops from high to medium in 5% manuring conditions. This conforms to the expectation of an aridity effect, and is also supported by previous research (Luo et al., 2018). However, in a pot growing experiment on *Setaria*, Lightfoot et al. (2020) found higher nitrogen isotope values with higher watering levels after excluding waterlogged plants. In their study the lowest watering level was 100 ml in 1 L pot every two days, somewhat higher than our lowest watering level of 34 ml in 0.5 L pot every day. Meanwhile, the study applied a lower temperature regime (28°C/22°C) than our study (30°C/28°C). It is possible that all the plants grown in the study of Lightfoot et al. (2020) were above the aridity threshold for *Setaria* with nitrogen isotope values affected by other factors than water stress.

There are variations in stable nitrogen isotope values among the three replicated plants of the same growing condition. Some plants have relatively consistent values (with a range of about or within 1 ‰), while others have a rather large range (up to 3.8 ‰). Because of the relatively small number of replicates in the current experiment, it is not clear whether large ranges are due to genetic variations among plants or just random factors. Lightfoot et al. (2020) found a range of 2.5–4.4 ‰ in the nitrogen isotope values of *Setaria* grown under each watering regime with more replicates (n=7–11) and multiple accessions. Experiments with a larger number of replicates of the same accession are needed to assess the variability in stable nitrogen isotope values of *Setaria* and *Panicum*.

Plants grown in the current experiment generally have high stable nitrogen isotope values. We did not measure the *δ* ^15^N values of the manure applied. The high isotope values may reflect a combination of the high temperature regime applied in the growth chamber and the likely high nitrogen isotope values of the manure we used (left to decompose for 12 months before application). During storage, significant quantities of nitrogen could have been lost from the manure through denitrification and ammonia volatilization. The nitrogen lost in this way is depleted in ^15^N and the nitrogen retained in the decomposing manure becomes enriched in ^15^N. Since plants grew in a soil-free (and thus nitrogen-free) medium, the millets relied solely on manure nitrogen, likely with a high ^15^N concentration (Bateman and Kelly, 2007). Combined with the high temperature regime in the glasshouse, this could explain why the nitrogen isotopic values of the millet grains in current study were at a higher level than that found in our previous study (5.8 to 6.3 ‰) with anaerobically stored cattle slurry grown under NW European field conditions (Christensen et al., 2022).

### 4.3 Charring effects

**Figure 2:**
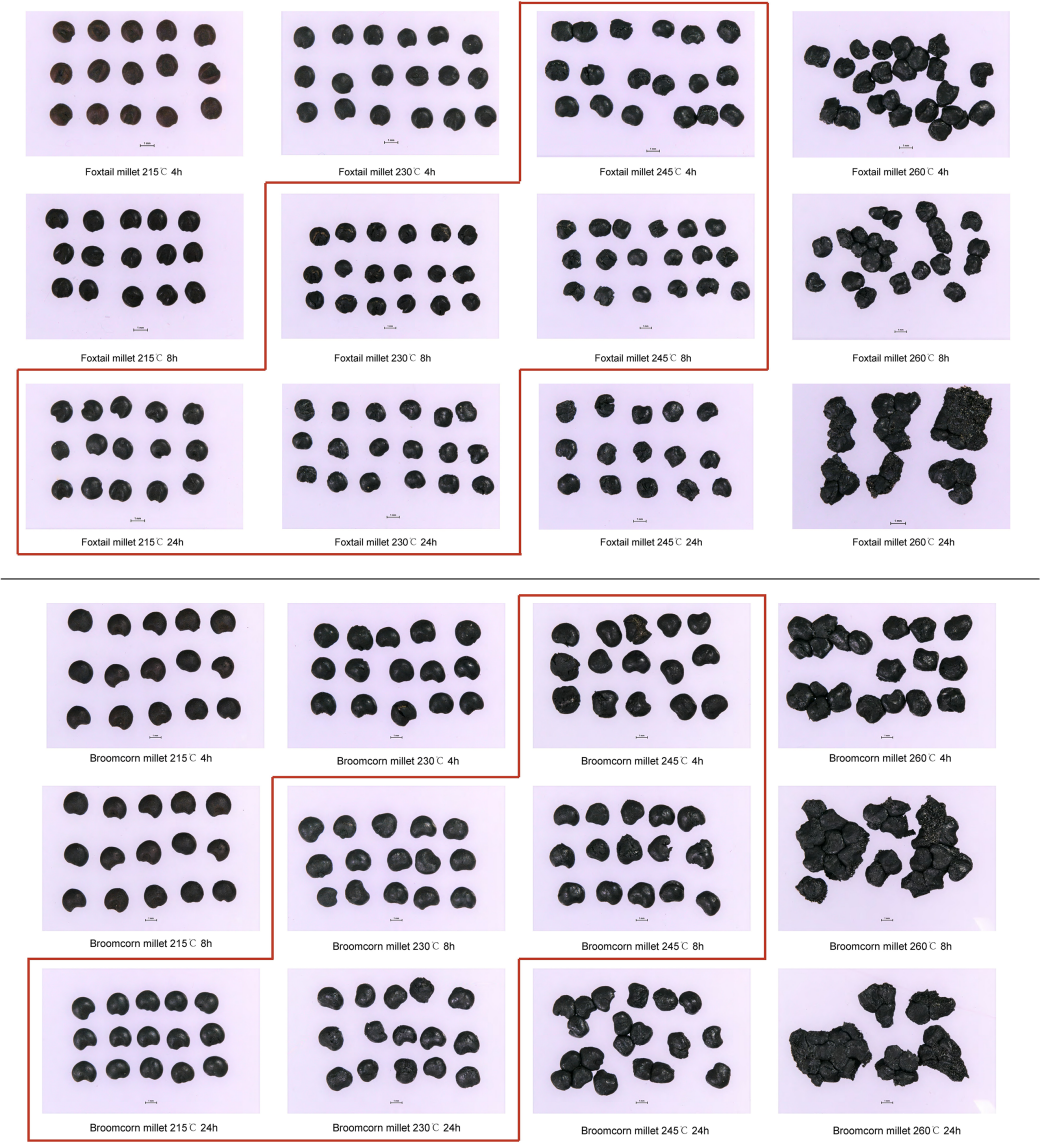
Charred foxtail (upper, *Setaria italica*) and broomcorn millet (lower, *Panicum miliaceum*) seeds under different charring conditions. The red box approximately delineates seeds comparable to well preserved archaeological remains.

Grains of foxtail and broomcorn millets, charred at 215 °C to 260 °C for 4 to 24 hrs, retained their physical morphology to varying degrees (**Figure 2**). When heated to 215 °C, the grains turned brown after 4 to 8 hours, but did not turn black. After 24 hrs the grains turned completely black but remained morphologically intact. When heated to 230 °C, *Panicum* grains showed slight distortion after 4 hrs, while *Setaria* grains did so after 8 hrs. When heated to 245 °C for 24 hrs, more than half of the grains were no longer identifiable. All grain samples were severely distorted when heated to 260 °C, with *Panicum* grains generally distorted more severely than *Setaria* grains. Grains of different varieties could have minor variations in response to heat. Previous research by Yang et al. (2011) also suggested that foxtail and broomcorn millets with identifiable morphological traits are probably charred between 200 and 250 °C.

Foxtail and broomcorn millets lost 13% and 15% of their original mass, respectively, when heated to 215°C for 4 hrs. The weight loss increased as temperature and duration increased; the loss was 67% and 66% of original mass when heated at 260°C for 24 hrs (**Table 4**). The carbon and nitrogen concentration of *Setaria* and *Panicum* grains also increased as temperature and duration increased (**Table 4**). The nitrogen concentration increased from ca. 2% (unheated) to 4– 5% (heated at 260°C for 24 hrs). Considering the mass loss of up to 67%, it is likely that there is minor nitrogen loss in heating (about 20–30% was lost). In contrast, carbon concentration only increased from 38% to 55% for *Setaria*, and 38% to 53% for *Panicum*, suggesting that a significant amount of carbon was lost during heating (about 50% were lost). It should also be noted that the highest carbon concentration is not in samples heated at 260°C for 24 hrs, but at 260°C for 8 hrs for *Setaria* and at 260°C for 4 hrs for *Panicum*, respectively. After these turning points, the carbon concentration drops again. It is likely that significant carbon loss occurred thereafter.

**Table 4:** Average weight loss, *δ* ^13^C, %C, *δ* ^15^N and %N of foxtail (*Setaria*) and broomcorn millet (*Panicum*) grains heated at different temperatures and durations.

Taxon	Temp (°C)	Time (hrs)	Weight Loss (%)	*δ* ^13^C (‰)	1σ	C%	*δ* ^15^N (‰)	1σ	N%
*Setaria*	50	48	0	–11.6	0.08	38.2	0.1	0.04	1.5
	215	4	13.2	–11.5	0.04	39.4	0.5	0.18	1.7
		8	17.1	–11.4	0.13	40.6	0.7	0.32	1.6
		24	27.7	–11.4	0.04	44.2	0.9	0.23	1.9
	230	4	15.4	–11.5	0.16	40.1	0.5	0.28	1.7
		8	20.6	–11.4	0.03	41.3	0.9	0.16	1.7
		24	46.9	–11.3	0.18	52.8	1.0	0.31	2.6
	245	4	32.0	–11.4	0.04	47.0	1.0	0.22	2.1
		8	46.2	–11.3	0.03	53.2	1.3	0.21	2.5
		24	59.0	–11.1	0.10	51.1	1.6	0.13	3.1
	260	4	45.5	–11.4	0.05	53.7	1.6	0.19	2.5
		8	53.8	–11.3	0.11	54.6	1.5	0.15	2.7
		24	67.3	–11.1	0.09	49.3	1.7	0.24	3.8
*Panicum*	50	48	0	–12.5	0.16	37.9	0.2	0.24	2.0
	215	4	16.9	–12.6	0.15	38.9	0.6	0.12	2.2
		8	18.7	–12.5	0.02	39.4	1.0	0.19	2.2
		24	31.0	–12.4	0.05	43.3	1.4	0.20	2.6
	230	4	18.2	–12.4	0.09	39.6	0.9	0.01	2.2
		8	34.2	–12.4	0.06	46.2	1.3	0.05	2.7
		24	50.2	–12.3	0.02	51.5	1.5	0.32	3.3
	245	4	35.8	–12.3	0.07	46.5	1.5	0.13	2.7
		8	47.6	–12.3	0.04	51.8	1.9	0.21	3.3
		24	59.2	–12.1	0.03	51.3	1.9	0.18	4.2
	260	4	44.6	–12.3	0.05	52.7	1.6	0.11	3.2
		8	52.1	–12.2	0.15	52.5	1.8	0.10	4.1
		24	65.9	–12.1	0.15	49.9	2.0	0.12	4.5

**Figure 3:**
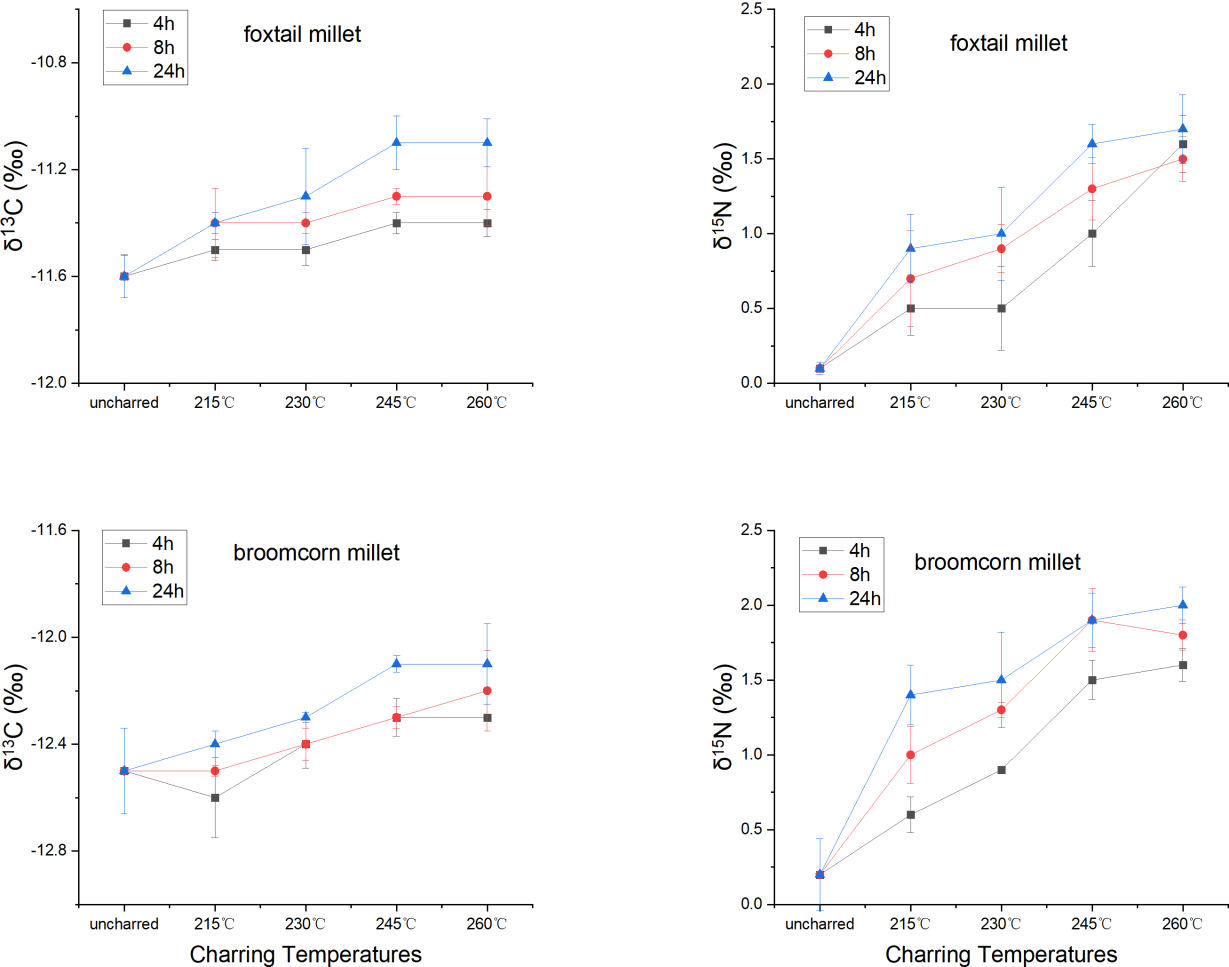
The change of carbon and nitrogen isotope values of foxtail (*Setaria italica*) and broomcorn millet (*Panicum miliaceum*) grains when heated at different temperatures and durations.

The stable carbon isotope values of charred *Setaria* and *Panicum* grains shifted 0.1–0.5 ‰ and –0.4–0 ‰, respectively, compared to uncharred grains (50°C for 48 hrs), while the stable nitrogen isotope values shifted 0.4–1.6 ‰ and 0.4–1.8 ‰ for grains when charred at different temperatures and durations (**Table 4**; **Table S2**). Generally, the shifts increased with higher temperature and longer duration. However, the carbon isotope values of *Setaria* and *Panicum* changed little (**Figure 3**). Samples heated at 215°C for 4 and 8 hrs and at 230°C for 4 hrs showed incomplete charring, and many samples heated at 245°C for 24 hrs or at 260°C for any duration were no longer identifiable morphologically. Therefore, only samples heated at 215°C for 24 hrs, 230°C for 8 and 24 hrs, and 245°C for 4 and 8 hrs are considered in estimating offsets between charred and uncharred grains (**Figure 2**).

The offsets of stable carbon and nitrogen isotope values in identifiable *Setaria* grains are 0.2–0.3 ‰ (mean 0.24 ‰) and 0.8–1.2 ‰ (mean 1.0 ‰), respectively. The offsets in identifiable *Panicum* grains are 0.1–0.2 ‰ (mean 0.16 ‰) and 1.1–1.7 ‰ (mean 1.3 ‰), respectively. The offset in stable carbon isotope values of both millets are minimal in the current study, consistent with previous studies on foxtail millet (Yang et al., 2011), broomcorn millet (Fraser et al., 2013), and several other crops (Fraser et al., 2013; Nitsch et al., 2015). The offsets in stable nitrogen isotope values of *Setaria* and *Panicum* observed in the current study are comparable to that of broomcorn millet in the previous study by Fraser et al. (2013), though higher than that of several crops including C_4_ pearl millet in other charring experiments (Nitsch et al., 2015; Styring et al., 2019).

### 4.4 The potential of stable isotope analysis of charred *Setaria* and *Panicum* remains for investigating ancient farming systems

Based on previous work and the current study, the application of manure leads to higher nitrogen isotope values of millets, and the magnitude of the shift is comparable for the two kinds of millets and wheat (**Figure 1A**). Hence, analyses of stable nitrogen isotope values represent an important tool in the study of prehistoric manuring in East Asian millets. Furthermore, the higher stable nitrogen isotope values of archaeological human remains, previously attributed to the consumption of large amounts of animal products, need to be reconsidered in light of the possible contributions of manured millets to people’s diet (Dong et al., 2021). As aridity may also lead to higher nitrogen isotope values of millets as in C_3_ plants, any environmental effects should be considered when interpreting isotopic signatures of archaeological remains.

Watering can cause a shift in stable carbon isotope values of C_4_ millets as well (**Figure 1B**), but the magnitude of the shift is much smaller than that of wheat and is close to the magnitude of analysis uncertainty. In the current study, stable carbon isotope values of both millets shifted 0.5 ‰ while the carbon isotope values of wheat shifted 1.4 ‰ from low watering to high watering levels under the 1% manure condition. Using stable carbon isotope values of millets to infer water status appears to be a challenge due to the small shift upon watering. The relatively large variations among different varieties pose additional complications (Lightfoot et al., 2016).

Our charring experiment demonstrated that the stable carbon and nitrogen isotope values of charred foxtail and broomcorn millet grains increased slightly from uncharred grains (**Figure 3**). The exact shift of isotopic values upon charring can vary depending on many factors, such as the variety (Charles et al., 2015), growing conditions (Yang et al., 2011), and ripeness and associated chemical composition of the grains. No significant change in stable carbon isotope values of foxtail and broomcorn millet grains was found in the current study. The current study suggests an increase of 1–2 ‰ in stable nitrogen isotope values of charred foxtail millet and broomcorn millet due to charring. This aligns with previous studies on grains from C_3_ crops (Fraser et al., 2013; Styring et al., 2013). It is noted, however, that other studies on C_3_ crops show smaller or non-systematic effect of charring on grain *δ* ^15^N values, regardless of grain size and degree of oxygen access during charring (Kanstrup et al., 2012; Nitsch et al., 2015).

## 5 Conclusions

The current study demonstrates that stable nitrogen isotope values of both foxtail and broomcorn millet seeds are positively correlated with manuring levels, making it feasible to use seed nitrogen isotopes values to identify past manuring practices, in agreement with recent field experimental results (Christensen et al., 2022). The stable nitrogen isotope values of both millets also increased as watering level decreased in the current study, showing an aridity effect similar to C_3_ crops. However, the opposite trend was found in foxtail millet in a previous study by Lightfoot et al. (2020). For stable carbon isotope values, the two millets exhibited opposing trends as watering level increased. The magnitude of these shifts in millet seed carbon isotope values is also smaller than that observed in wheat, making it difficult to identify the watering status of millets archaeologically. Charring can lead to increases up to 1–2 ‰ in stable nitrogen isotope values of both millets, while the carbon isotope values of foxtail and broomcorn millet change less than 0.3 ‰. Overall, we demonstrated that stable nitrogen isotope values of charred foxtail and broomcorn millet seeds could provide insight into past field management practices, while both carbon and nitrogen isotope values can inform palaeodietary reconstruction. However, to investigate growing conditions and manure management in prehistoric agriculture, more information would be beneficial regarding the type, quantity and quality of historic manures, the frequency of application, and how manure was stored before application to crops.

## Data availability statement

The original contributions presented in the study are included in the article/**Supplementary Material**. Further inquiries can be directed to the corresponding author.

## Author contributions

YD and AB conceived and planned the experiments. YD, XB, and RW carried out the experiments. EB, NH, and BC contributed to sample preparation. YD, XB, RW, BC, MC, and AB contributed to the interpretation of the results. YD wrote the first draft of the manuscript. All authors contributed to manuscript revision, read, and approved the submitted version.

## Funding

This project was funded by the Visiting Fellowship of British Academy (VF1_100658), Shandong Provincial Natural Science Foundation (ZR2022MD111), Shandong University (2020QNQT018), China Scholarship Council, and the University of Oxford's John Fell Fund.

## Acknowledgment

We thank Dr. Peter Ditchfield for helping us to analyse the samples in the stable isotope laboratory, School of Archaeology, University of Oxford. We are also grateful for two reviewers and the editor who have provided many constructive comments and greatly improved the manuscript.

## Conflict of interest

The authors declare that the research was conducted in the absence of any commercial or financial relationships that could be construed as a potential conflict of interest.

## Publisher’s note

All claims expressed in this article are solely those of the authors and do not necessarily represent those of their affiliated organizations, or those of the publisher, the editors and the reviewers. Any product that may be evaluated in this article, or claim that may be made by its manufacturer, is not guaranteed or endorsed by the publisher.
