# Supplementary material for: The potential of stable carbon and nitrogen isotope analysis of foxtail and broomcorn millets for investigating ancient farming systems
Source: Front Plant Sci. 2022 Oct 19;13:1018312. doi: 10.3389/fpls.2022.1018312 (PMC9627502; doi:10.3389/fpls.2022.1018312)
Supplement: Supplementary file 1 [file DataSheet_1.docx]

**Supplementary Information**

The potential of stable carbon and nitrogen isotope analysis of foxtail and broomcorn millets for investigating ancient farming systems

Yu Dong^1, 2, *^, Xiaoguang Bi^1^, Rubi Wu^2^, Eric J. Belfield^3^, Nicholas P. Harberd^3^, Bent T. Christensen^4^, Mike Charles^2^, Amy Bogaard^2^

1. Institute of Cultural Heritage, Shandong University, Qingdao, Shandong 266237, P. R. China

2. School of Archaeology, University of Oxford, Oxford OX1 3TG, United Kingdom

3. Department of Plant Sciences, University of Oxford, Oxford OX1 3RB, United Kingdom

4. Department of Agroecology, Aarhus University, AU-Foulum, Blichers Allé 20, DK-8830 Tjele, Denmark

^*^Corresponding author. *E-mail address:* [yudong@sdu.edu.cn](mailto:yudong@sdu.edu.cn)

**Table S1.** The carbon and nitrogen isotope values and contents of foxtail millet (*Setaria*), broomcorn millet (*Panicum*), and wheat (*Triticum*) grains grown under different manuring and watering levels.

| Taxon | Manuring Levels | Watering Levels | Sample ID | *δ*^13^C (‰) | C% | *δ*^15^N (‰) | N% |
| --- | --- | --- | --- | --- | --- | --- | --- |
| *Setaria* | 1% | low | SET030 | -12.1 | 42.1 | 14.1 | 1.7 |
|  |  |  | SET033 | -11.9 | 41.3 | 13.6 | 1.7 |
|  |  |  | SET033_rep | -11.9 | 40.5 |  |  |
|  |  |  | SET038 | -12.7 | 40.1 | 10.3 | 2.3 |
|  |  | medium | SET055 | -12.3 | 41.8 | 10.4 | 1.8 |
|  |  |  | SET064 | -11.7 | 39.7 | 11.2 | 1.9 |
|  |  |  | SET064_rep | -11.7 | 41.5 |  |  |
|  |  |  | SET065 | -12.2 | 41.3 | 8.2 | 2.7 |
|  |  |  | SET065_rep |  |  | 8.5 | 2.7 |
|  |  | high | SET045 | -11.8 | 40.5 | 11.3 | 2.0 |
|  |  |  | SET047 | -11.8 | 46.0 | 10.4 | 1.9 |
|  |  |  | SET051 | -11.7 | 44.9 | 11.8 | 1.7 |
|  | 5% | medium | SET052 | -12.4 | 44.5 | 16.4 | 1.9 |
|  |  |  | SET053 | -12.4 | 43.7 | 15.2 | 2.0 |
|  |  |  | SET054 | -12.4 | 41.3 | 15.9 | 1.7 |
|  |  | high | SET007 | -12.0 | 45.4 | 14.3 | 2.8 |
|  |  |  | SET012 | -13.6 | 43.1 | 16.1 | 2.4 |
|  |  |  | SET024 | -12.2 | 41.0 | 12.8 | 1.7 |
| *Panicum* | 1% | low | PAN001 | -13.5 | 40.5 | 14.4 | 1.6 |
|  |  |  | PAN008 | -13.6 | 44.8 | 15.4 | 1.6 |
|  |  |  | PAN009 | -13.6 | 46.5 | 14.3 | 2.1 |
|  |  | medium | PAN017 | -14.0 | 39.5 | 13.4 | 1.9 |
|  |  |  | PAN018 | -13.9 | 37.1 | 13.3 | 1.8 |
|  |  | high | PAN010 | -14.2 | 43.8 | 10.9 | 1.8 |
|  |  |  | PAN010_rep | -14.1 | 44.9 | 11.1 | 1.7 |
|  |  |  | PAN016 | -14.2 | 40.4 | 12.1 | 2.5 |
|  | 5% | medium | PAN019 | -13.7 | 43.0 | 15.7 | 1.6 |
|  |  |  | PAN020 | -13.7 | 38.3 | 14.6 | 1.5 |
|  |  | high | PAN026 | -13.9 | 41.2 | 16.0 | 1.8 |
|  |  |  | PAN027 | -14.0 | 39.9 | 13.5 | 1.6 |
|  |  |  | PAN027_rep | -14.0 | 39.4 |  |  |
|  |  |  | PAN028 | -13.8 | 38.8 | 14.8 | 1.7 |
| *Triticum* | 1% | low | WHE001 | -28.6 | 46.4 | 12.5 | 3.3 |
|  |  |  | WHE002 | -29.2 | 42.5 | 13.2 | 3.2 |
|  |  |  | WHE003 | -29.3 | 44.2 | 13.8 | 3.7 |
|  |  | medium | WHE007 | -28.7 | 42.9 | 10.0 | 3.5 |
|  |  |  | WHE008 | -29.0 | 42.7 | 11.2 | 3.7 |
|  |  |  | WHE009 | -29.2 | 43.8 | 11.3 | 3.3 |
|  |  |  | WHE009_rep |  |  | 11.8 | 3.3 |
|  |  | high | WHE004 | -30.8 | 42.3 | 11.9 | 3.4 |
|  |  |  | WHE005 | -30.0 | 40.0 | 11.3 | 3.4 |
|  |  |  | WHE006 | -30.4 | 42.5 | 10.9 | 3.1 |
|  |  |  | WHE006_rep | -30.3 | 43.9 |  |  |
|  | 5% | medium | WHE010 | -30.7 | 41.5 | 15.2 | 3.9 |
|  |  |  | WHE011 | -29.7 | 40.9 | 18.2 | 4.1 |
|  |  | high | WHE012 | -30.2 | 42.2 | 15.4 | 3.9 |
|  |  |  | WHE013 | -31.4 | 42.2 | 16.4 | 3.8 |

**Table S2.** The *δ*^13^C, %C, *δ*^15^N and %N values of foxtail (*Setaria*) and broomcorn millet (*Panicum*) grains heated at different temperatures and durations.

| Taxon | Temp (℃) | Time (hrs) | Sample ID | *δ*^13^C (‰) | C% | *δ*^15^N (‰) | N% |
| --- | --- | --- | --- | --- | --- | --- | --- |
| *Setaria* | 50 | 48 | UNFM01 | -11.7 | 38.0 | 0.1 | 1.6 |
|  |  |  | UNFM02 | -11.5 | 38.3 | 0.2 | 1.5 |
|  |  |  | UNFM03 | -11.6 | 38.3 | 0.1 | 1.5 |
|  | 215 | 4 | F215-4A | -11.5 | 39.6 | 0.7 | 1.6 |
|  |  |  | F215-4B | -11.5 | 39.4 | 0.4 | 1.7 |
|  |  |  | F215-4B_rep | -11.5 | 39.4 | 0.3 | 1.6 |
|  |  |  | F215-4C | -11.6 | 39.3 | 0.5 | 1.8 |
|  |  | 8 | F215-8A | -11.4 | 40.6 | 0.9 | 1.6 |
|  |  |  | F215-8B | -11.3 | 40.8 | 0.3 | 1.5 |
|  |  |  | F215-8C | -11.5 | 40.4 | 0.9 | 1.8 |
|  |  | 24 | F215-24A | -11.4 | 42.7 | 0.7 | 1.8 |
|  |  |  | F215-24B | -11.4 | 44.2 | 0.9 | 1.9 |
|  |  |  | F215-24C | -11.3 | 45.6 | 1.2 | 1.9 |
|  | 230 | 4 | F230-4A | -11.3 | 40.3 | 0.5 | 1.6 |
|  |  |  | F230-4B | -11.6 | 40.2 | 0.7 | 1.7 |
|  |  |  | F230-4C | -11.6 | 39.8 | 0.2 | 2.0 |
|  |  | 8 | F230-8A | -11.4 | 41.2 | 1.0 | 1.5 |
|  |  |  | F230-8A_rep | -11.4 | 41.4 | 1.0 | 1.5 |
|  |  |  | F230-8B | -11.4 | 41.3 | 1.1 | 1.7 |
|  |  |  | F230-8C | -11.4 | 41.5 | 0.7 | 1.8 |
|  |  | 24 | F230-24A | -11.5 | 53.6 | 1.3 | 2.8 |
|  |  |  | F230-24B | -11.3 | 53.7 | 1.2 | 2.5 |
|  |  |  | F230-24C | -11.1 | 51.0 | 0.7 | 2.4 |
|  | 245 | 4 | F245-4A | -11.4 | 47.9 | 0.9 | 2.1 |
|  |  |  | F245-4B | -11.3 | 46.9 | 0.8 | 2.1 |
|  |  |  | F245-4C | -11.4 | 46.2 | 1.3 | 2.1 |
|  |  | 8 | F245-8A | -11.2 | 53.3 | 1.1 | 2.5 |
|  |  |  | F245-8B | -11.3 | 53.5 | 1.5 | 2.5 |
|  |  |  | F254-8C | -11.3 | 53.0 | 1.4 | 2.5 |
|  |  |  | F245-8C_rep | -11.3 | 52.9 | 1.5 | 2.5 |
|  |  | 24 | F245-24A | -11.2 | 51.6 | 1.4 | 3.2 |
|  |  |  | F245-24B | -11.0 | 51.3 | 1.7 | 3.1 |
|  |  |  | F245-24C | -11.2 | 50.5 | 1.6 | 3.0 |
|  | 260 | 4 | F260-4A | -11.5 | 55.4 | 1.3 | 2.7 |
|  |  |  | F260-4B | -11.4 | 52.5 | 1.6 | 2.4 |
|  |  |  | F260-4C | -11.4 | 53.0 | 1.7 | 2.4 |
|  |  | 8 | F260-8A | -11.1 | 54.7 | 1.4 | 2.8 |
|  |  |  | F260-8B | -11.3 | 54.7 | 1.6 | 2.7 |
|  |  |  | F260-8C | -11.3 | 54.3 | 1.3 | 2.7 |
|  |  | 24 | F260-24A | -11.0 | 50.4 | 1.7 | 3.9 |
|  |  |  | F260-24B | -11.2 | 49.6 | 2.0 | 3.8 |
|  |  |  | F260-24C | -11.0 | 49.6 | 1.4 | 3.9 |
|  |  |  | F260-24C_rep | -11.0 | 47.9 | 1.5 | 3.7 |
| *Panicum* | 50 | 48 | UNCM01 | -12.5 | 37.7 | 0.4 | 1.9 |
|  |  |  | UNCM02 | -12.7 | 37.8 | 0.0 | 1.9 |
|  |  |  | UNCM03 | -12.4 | 38.2 | 0.3 | 2.3 |
|  | 215 | 4 | C215-4A | -12.5 | 39.1 | 0.5 | 2.1 |
|  |  |  | C215-4B | -12.5 | 39.1 | 0.7 | 2.2 |
|  |  |  | C215-4C | -12.8 | 38.6 | 0.7 | 2.3 |
|  |  | 8 | C215-8A | -12.5 | 39.8 | 1.0 | 2.2 |
|  |  |  | C215-8B | -12.5 | 39.4 | 0.9 | 2.1 |
|  |  |  | C215-8C | -12.5 | 39.1 | 1.2 | 2.3 |
|  |  | 24 | C215-24A | -12.4 | 43.0 | 1.4 | 2.6 |
|  |  |  | C215-24B | -12.4 | 43.8 | 1.1 | 2.6 |
|  |  |  | C215-24B_rep | -12.4 | 43.9 | 1.1 | 2.7 |
|  |  |  | C215-24C | -12.5 | 43.2 | 1.5 | 2.6 |
|  | 230 | 4 | C230-4A | -12.3 | 39.9 | 0.9 | 2.2 |
|  |  |  | C230-4B | -12.4 | 40.5 | 0.9 | 2.2 |
|  |  |  | C230-4C | -12.5 | 38.5 | 0.9 | 2.2 |
|  |  | 8 | C230-8A | -12.4 | 48.1 | 1.3 | 2.9 |
|  |  |  | C230-8B | -12.3 | 44.2 | 1.3 | 2.5 |
|  |  |  | C230-8C | -12.5 | 46.5 | 1.2 | 2.7 |
|  |  | 24 | C230-24A | -12.3 | 50.3 | 1.8 | 3.5 |
|  |  |  | C230-24B | -12.3 | 52.3 | 1.2 | 3.2 |
|  |  |  | C230-24C | -12.3 | 51.9 | 1.6 | 3.3 |
|  | 245 | 4 | C245-4A | -12.4 | 46.3 | 1.5 | 2.8 |
|  |  |  | C245-4A_rep | -12.4 | 46.0 | 1.5 | 2.8 |
|  |  |  | C245-4B | -12.4 | 46.2 | 1.5 | 2.7 |
|  |  |  | C245-4C | -12.3 | 46.8 | 1.3 | 2.7 |
|  |  | 8 | C245-8A | -12.3 | 52.0 | 2.0 | 3.2 |
|  |  |  | C245-8B | -12.3 | 51.2 | 2.0 | 3.4 |
|  |  |  | C245-8C | -12.4 | 52.2 | 1.6 | 3.4 |
|  |  | 24 | C245-24A | -12.2 | 52.3 | 1.7 | 4.0 |
|  |  |  | C245-24B | -12.1 | 51.0 | 2.1 | 4.4 |
|  |  |  | C245-24C | -12.1 | 50.5 | 1.9 | 4.1 |
|  | 260 | 4 | C260-4A | -12.3 | 53.3 | 1.5 | 3.3 |
|  |  |  | C260-4B | -12.4 | 52.4 | 1.7 | 3.2 |
|  |  |  | C260-4C | -12.3 | 52.6 | 1.7 | 3.1 |
|  |  |  | C260-4C_rep | -12.3 | 52.3 | 1.6 | 3.1 |
|  |  | 8 | C260-8A | -12.3 | 53.7 | 1.8 | 4.0 |
|  |  |  | C260-8B | -12.3 | 54.1 | 1.7 | 3.5 |
|  |  |  | C260-8C | -12.0 | 49.6 | 1.9 | 4.9 |
|  |  | 24 | C260-24A | -12.0 | 50.0 | 2.1 | 5.2 |
|  |  |  | C260-24B | -12.1 | 50.2 | 1.9 | 4.9 |
|  |  |  | C260-24C | -12.3 | 49.6 | 2.0 | 3.6 |

Table S3. Standard deviations for the carbon and nitrogen isotopic compositions of the calibration standards used in each analytical session associated with the data presented in this paper.

| **Standard** | **Run ID ^i^** | ***n*** | ***δ*^13^C (±1σ, ‰)** | ***δ*^15^N (±1σ, ‰)** |
| --- | --- | --- | --- | --- |
| IRM-1 ^ii^ | RLAHA_181217 | 14 | 0.05 | − |
| USGS41 | RLAHA_181217 | 7 | 0.19 | − |
| IRM-1 | RLAHA_190116 | 10 | 0.05 |  |
| USGS41 | RLAHA_190116 | 5 | 0.03 |  |
| IRM-1 | RLAHA_190121 | 9 |  | 0.25 |
| IRM-2 ^iii^ | RLAHA_190121 | 6 |  | 0.30 |
| IRM-1 | RLAHA_190123 | 4 |  | 0.68 |
| IRM-2 | RLAHA_190123 | 2 |  | 0.49 |
| USGS40 | RLESA_20220118 | 3 | 0.03 | 0.03 |
| USGS41a | RLESA_20220118 | 3 | 0.05 | 0.18 |
| USGS62 | RLESA_20220118 | 3 | 0.04 | 0.02 |

i. Runs with an ID starting with RLAHA were analyzed at the Research Laboratory for Archaeology and the History of Art (RLAHA), University of Oxford, Runs with an ID starting with RLESA were analyzed at the Joint International Research Laboratory of Environmental and Social Archaeology (JoInRLESA), Shandong University.

ii. IRM-1 is an internal standard, the material is alanine.

iii. IRM-2 is an internal standard, the material is seal collagen.

**Table S4.** Standard reference materials used to monitor internal accuracy and precision.

| Standard | Material | Mean *δ*^13^C  (‰, VPDB) | Mean *δ*^15^N  (‰, AIR) |
| --- | --- | --- | --- |
| IAEA-CH-6 | Sucrose | −10.449±0.033 | − |
| EMA B2159 | Sorghum | -13.68±0.18 | 1.58±0.17 |

VPDB stands for Vienna Pee Dee Belemnite; AIR stands for Ambient Inhalable Reservoir.

Table S5. Mean and standard deviations of all the check (QA) standards analyzed in the analytical sessions associated with data presented in this paper.

|  |  |  | ***δ*^13^C (‰, VPDB)** | | | ***δ*^15^N (‰, AIR)** | | |
| --- | --- | --- | --- | --- | --- | --- | --- | --- |
| **Standard** | **Run ID ^*^** | ***n*** | **Mean** | **±** | **1σ** | **Mean** | **±** | **1σ** |
| IAEA-CH-6 | RLAHA_181217 | 7 | -10.15 | ± | 0.03 |  |  |  |
| IAEA-CH-6 | RLAHA_190116 | 5 | -10.20 | ± | 0.04 |  |  |  |
| EMA B2159 | RLAHA_190121 | 6 |  |  |  | 1.65 | ± | 0.46 |
| EMA B2159 | RLAHA_190123 | 2 |  |  |  | 0.73 | ± | 0.34 |
| EMA B2159 | RLESA_20220118 | 12 | -13.44 | ± | 0.09 | 1.67 | ± | 0.12 |

* Runs with an ID starting with RLAHA were analyzed at the Research Laboratory for Archaeology and the History of Art, University of Oxford, Runs with an ID starting with RLESA were analyzed at the Joint International Research Laboratory of Environmental and Social Archaeology, Shandong University.

**Table S6.** Carbon and nitrogen isotope compositions of samples analyzed in duplicate.

|  | ***δ*^13^C (‰)** | | ***δ*^15^N (‰)** | | ***Analysis Lab^*^*** |
| --- | --- | --- | --- | --- | --- |
| **Sample ID** | **A** | **B** | **A** | **B** |  |
| PAN010 | -14.09 | -14.20 |  |  | RLAHA，University of Oxford |
| PAN018 | -13.93 | -13.94 |  |  | RLAHA，University of Oxford |
| PAN027 | -14.04 | -14.01 |  |  | RLAHA，University of Oxford |
| WHE006 | -30.40 | -30.34 |  |  | RLAHA，University of Oxford |
| WHE014 | -25.54 | -25.54 |  |  | RLAHA，University of Oxford |
| SET033 | -11.85 | -11.85 |  |  | RLAHA，University of Oxford |
| SET064 | -11.73 | -11.72 |  |  | RLAHA，University of Oxford |
| PAN010 |  |  | 10.89 | 11.15 | RLAHA，University of Oxford |
| SET038 |  |  | 9.80 | 10.11 | RLAHA，University of Oxford |
| SET065 |  |  | 8.22 | 8.52 | RLAHA，University of Oxford |
| WHE009 |  |  | 11.25 | 11.26 | RLAHA，University of Oxford |
| C215-24B | -12.39 | -12.39 | 1.12 | 1.12 | JoInRLESA，Shandong University |
| C245-4A | -12.39 | -12.40 | 1.52 | 1.45 | JoInRLESA，Shandong University |
| C260-4C | -12.32 | -12.34 | 1.68 | 1.58 | JoInRLESA，Shandong University |
| F215-4B | -11.54 | -11.54 | 0.37 | 0.33 | JoInRLESA，Shandong University |
| F230-8A | -11.36 | -11.38 | 0.95 | 0.97 | JoInRLESA，Shandong University |
| F245-8C | -11.30 | -11.30 | 1.42 | 1.46 | JoInRLESA，Shandong University |
| F260-24C | -10.96 | -11.01 | 1.39 | 1.48 | JoInRLESA，Shandong University |
| UNCM03 | -12.39 | -12.47 | 0.27 | 0.25 | JoInRLESA，Shandong University |

* RLAHA stands for the Research Laboratory for Archaeology and the History of Art, and JoInRLESA stands for the Joint International Research Laboratory of Environmental and Social Archaeology.

**Standard Uncertainty**

Standard uncertainty for the *δ*^13^C and *δ*^15^N measurements of the samples was estimated following Szpak et al. (2017).

For analysis done at RLAHA, University of Oxford, the systematic errors (*u_(bias)_*) were calculated to be ±0.28‰ for *δ*^13^C and ±0.18‰ for *δ*^15^N. Random errors (*uR_(w)_*) were calculated to be ±0.09‰ for *δ*^13^C and ±0.36‰ for *δ*^15^N based on the pooled standard deviations of the check standards and sample replicates. Standard uncertainty was determined to be ±0.29‰ for *δ*^13^C and ±0.41‰ for *δ*^15^N.

However, one run (RLAHA_190123 which includes the measurement of nitrogen isotope values of wheat) done at RLAHA, University of Oxford, has relatively large systematic errors (*u_(bias)_*=0.87‰) and slightly big random errors (*uR_(w)_*= 0.59‰). We need to be cautious in interpreting the nitrogen isotope values of wheat in current study.

For analysis done at JoInRLESA, Shandong University, the systematic errors (*u_(bias)_*) were calculated to be ±0.30‰ for *δ*^13^C and ±0.19‰ for *δ*^15^N. Random errors (*uR_(w)_*) were calculated to be ±0.08‰ for *δ*^13^C and ±0.12‰ for *δ*^15^N based on the pooled standard deviations of the check standards and sample replicates. Standard uncertainty was determined to be ±0.31‰ for *δ*^13^C and ±0.22‰ for *δ*^15^N.

**References**

Szpak, P., Metcalfe, J. Z., and Macdonald, R. A. (2017). Best practices for calibrating and reporting stable isotope measurements in archaeology. *J. Archaeol. Sci. Reports* 13, 609–616. doi: 10.1016/j.jasrep.2017.05.007.
